# Sex differences in cognitive decline and impairment: a scoping review in informatics literature

**DOI:** 10.1186/s13293-025-00804-6

**Published:** 2025-12-16

**Authors:** Muskan Garg, Xingyi Liu, Jie Lin, Maria Vassilaki, Ronald C. Petersen, Jennifer St. Sauver, Ekta Kapoor, Sunghwan Sohn

**Affiliations:** 1 Department of Artificial Intelligence & Informatics, Mayo Clinic, Rochester, MN USA; 2 Department of Quantitative Health Sciences, Mayo Clinic, Rochester, MN USA; 3 Department of Neurology, Mayo Clinic, Rochester, MN USA; 4 Division of General Internal Medicine, Mayo Clinic, Rochester, MN USA; 5 Center for Women’s Health, Mayo Clinic, Rochester, MN USA

**Keywords:** Alzheimer’s disease, Cognitive impairment, Cognitive decline, Dementia, Sex disparities, Clinical decision support systems

## Abstract

**Objectives:**

A scoping review was conducted to investigate knowledge gaps in the informatics research literature regarding sex differences in cognitive decline and impairment, identifying existing studies and areas requiring further exploration.

**Methods and materials:**

Our scoping review follows the Preferred Reporting Items for Systematic reviews and Meta-Analysis Extension for Scoping Reviews (PRISMA – ScR) guidelines. We searched Ovid and other databases (APA PsychInfo, EMB Reviews, and Embase) for studies on sex differences in cognitive decline and impairment, focusing on peer-reviewed informatics journals and conference proceedings from 2000 to 2025. The selected manuscripts were analyzed based on metadata statistics, study attributes, and thematic content.

**Results:**

A total of 17 full articles met the inclusion criteria. Most studies were conducted in North America (*n* = 7) and the European Union (*n* = 5). More than half of the studies were published after 2020 (*n* = 10). Our analyses highlight key aspects of selected studies, including bibliometric metadata, study attributes (e.g., study types, methods, and data sources), and thematic findings. Statistical modeling (*n* = 8) and machine learning (*n* = 4) are the most widely used study methods. Majority (*n* = 11) of the publications are single-site studies, while the other multi-site collaborations (*n* = 6) have emerged among hospitals, academic institutions, and research institutions.

**Discussion:**

Sex-specific disparities in cognitive decline and impairment remain a critical issue in healthcare. Most informatics research has primarily concentrated on identifying generic sex differences in cognitive decline and impairment progression, rather than exploring the complex underlying mechanisms such as observational studies with causal analysis. While these studies are valuable, they lack a holistic approach to understanding sex-specific disparities.

**Conclusion:**

There is a significant gap in using informatics to understand how biological, social, and behavioral factors contribute to sex-specific disparities in cognitive decline and impairment. This limitation underscores the need for more comprehensive informatics research that goes beyond mere identification to find the root cause of these disparities in healthcare.

**Supplementary Information:**

The online version contains supplementary material available at 10.1186/s13293-025-00804-6.

## Plain language summary

This study looked at what researchers have found—and what they haven’t—about how cognitive decline (like memory loss or dementia) affects men and women differently. We focused on research in the field of health informatics, which uses data and technology to study health. We followed a standard review process and searched major medical and psychology databases. Out of everything we found, 17 studies fit our criteria. More than half of the studies were published after 2020, with most originating from the U.S.A. and Europe. We analyzed key statistics from the selected studies and found that researchers often used statistical models and machine learning techniques to identify differences between men and women in how cognitive decline develops. To present our findings clearly, we organized the results into three parts: (a) an overview of how research in this area has evolved over time, (b) a summary of study designs and data sources, and (c) an analysis of common themes and focus areas across the studies. Our review found that most studies simply highlight that there are differences between men and women in how cognitive decline occurs, but they rarely explore why these differences exist. There is very little research that examines the biological, social, or behavioral factors that may explain these patterns. This points to a clear need for future studies to go beyond identifying differences and focus on understanding the root causes behind them.

**Supplementary Information:**

The online version contains supplementary material available at 10.1186/s13293-025-00804-6.

## Highlights

- Examine the existing informatics literature on sex-specific differences for cognitive decline and impairment.
- Reported the metadata analysis to examine the bibliographic landscape of the research field and its development over time.
- Discussed attributes (types, methods and data) of past studies for contextual understanding.
- Performed thematic analysis (clinical, biomedical, and psychological & behavioral) for sex differences.
- Recommended future research directions for healthcare informatics research on sex-specific differences in cognitive decline and impairment.

**Supplementary Information:**

The online version contains supplementary material available at 10.1186/s13293-025-00804-6.

## Introduction

Around 6.2 million Americans aged 65 and above are diagnosed with Alzheimer’s dementia. Without significant medical advancements that can prevent, halt, or cure Alzheimer’s Disease (AD), this figure might double, reaching 13.8 million by 2060 [1, 2]. Existing studies demonstrate significant impacts on the quality of life due to cognitive decline, presenting substantial challenges to healthcare systems [3]. According to Alzheimer’s Research UK, 65% of individuals living with dementia are female. However, despite this higher prevalence, females are more likely to be underdiagnosed compared to males^1^. A cohort study suggests females may have a greater cognitive reserve (global cognition, executive function, and memory) but experience faster cognitive decline than males, contributing to sex differences in late-life dementia risk [4]. The Alzheimer’s Society suggests that implementing sex-specific cognitive assessments could help address these disparities and improve diagnostic accuracy. Thus, evidence-based, sex-specific informatics research can enhance healthcare decision-making, allocate resources more efficiently, optimize patient outcomes, and reduce misdiagnosis risks by tailoring interventions to the unique needs of both males and females [5].

Personalized medicine is becoming increasingly important in healthcare as the prevalence and progression of cognitive decline appear to differ between males and females [6]. Females with dementia face higher hospitalization rates due to falls and medication complications, yet their symptoms are often misattributed to aging [7, 8]. Additionally, caregiving dynamics differ, with females receiving more informal home care and males being institutionalized more often, influencing long-term health outcomes. Addressing these disparities is essential for developing more effective, equitable dementia care strategies.

Despite the significant scientific advances achieved so far, most current healthcare informatics methods do not account for healthcare disparities [5, 9]. The past review articles, indirectly associated with sex-specific disparities in cognitive decline, focussed on clinical trials and investigated sexual minority groups [4]. However, there is a notable gap in investigating the informatics literature-based study designs for discovering sex differences in cognitive decline.

Our scoping review aims to examine the existing informatics literature on sex-specific differences for cognitive decline and impairment. We limited our review to informatics venues (i.e., informatics journals and conference papers) to specially characterize how the informatics community has addressed sex differences in cognitive decline and impairment. The objective of this work was to identify and categorize existing studies to understand the breadth and depth of current research in informatics venues. It incorporates the characterization of existing studies. We highlight areas where informatics has successfully contributed to understanding these differences and identify gaps where further research is needed. This approach offers a unique lens through which we understand the need for advanced healthcare informatics in investigating sex-specific differences for better decision-making.

## Methods

We targeted publications on sex-specific differences in peer-reviewed informatics journals and conference proceedings published in English.

**Table 1:** The search strategy used in the scoping review

SN	Searches	Hits
1	*Concept A: Cognitive decline and impairment	1,283,605
2	*Concept B: Sex-specific terms	7,848,273
3	1 and 2	128,777
4	exp Healthcare Disparities/	55,783
5	*Concept C: Healthcare disparities terms	19,987,742
6	4 or 5	20,000,911
7	3 and 6	58,262
8	Informatics/or Medical Informatics/	53,571
9	information science/	14,471
10	*Concept D: Informatics literature terms	306,373
11	informatics.jw.	126,629
12	artificial intelligence in medicine.jn.	5338
13	“methods of information in medicine”.jn.	6309
14	“journal of medical internet research”.jn	27,893
15	amia.jw	13,013
16	Or/8–15	493,466
17	7 and 16	161
18	*Concept E: Genetics and proteomics terms	12,175,876
19	17 not 18	114
20	(exp animals/or exp nonhuman/) not (exp humans/or exp patient/)	13,154,229
21	*Concept F: Animal related studies	11,516,646
22	19 not (20 or 21)	114
23	*Concept G: Non-peer-reviewed studies	13
24	22 not 23	101
25	limit 24 to yr="2000 -Current”	101
26	remove duplicates from 25	54

*Details on concepts A-G are given in Appendix A

Based on this criterion, a senior librarian devised a searching strategy (see Table 1) for identifying articles from a set of databases. We systematically searched the following multiple databases for this scoping review: APA PsycInfo, EBM Reviews - Cochrane Central Register of Controlled Trials, EBM Reviews - Cochrane Database of Systematic Reviews, Embase, Ovid MEDLINE(R), Epub. All these datasets were retrieved for articles before 22 September 2025. In addition to this, we obtained 11 articles from SCOPUS database.

**Fig. 1:**
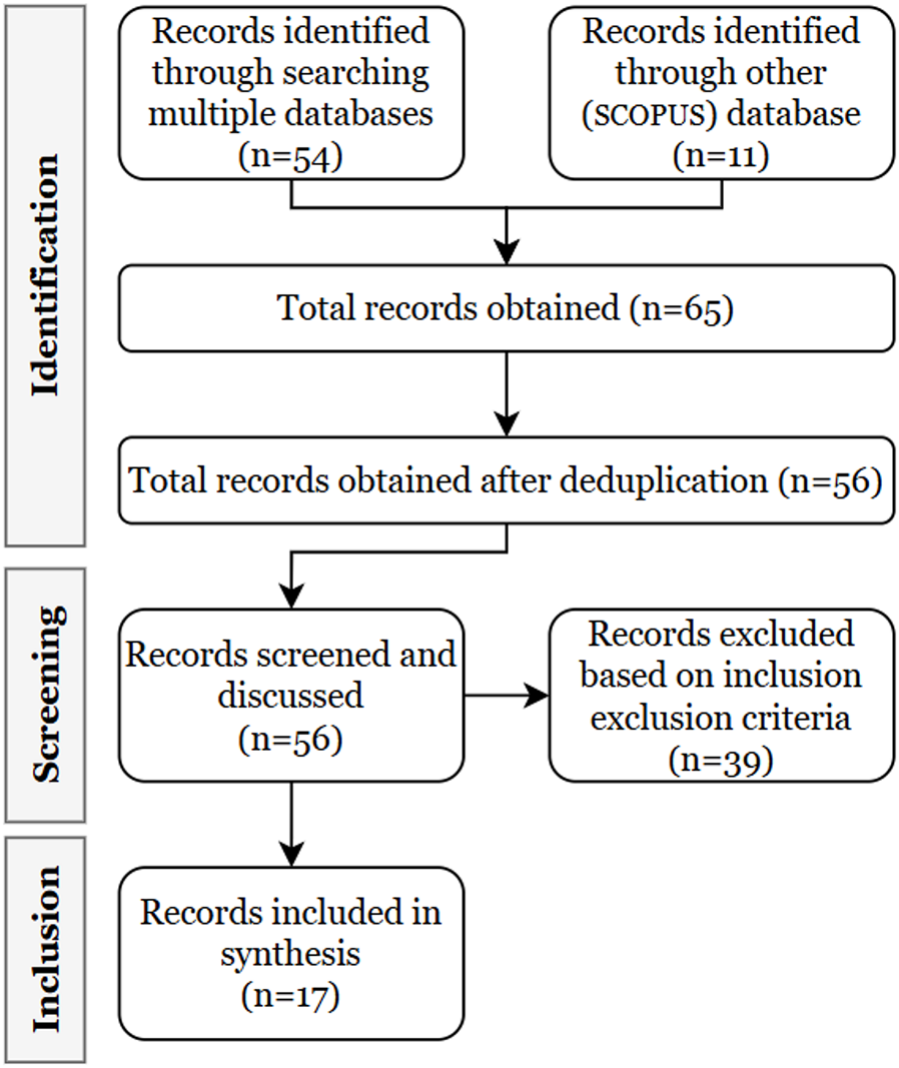
Article screening process

We first identified the articles with (i) healthcare sex-specific differences/disparities in cognitive decline and impairment, (ii) articles from informatics literature, and merged them to obtain records as shown in Fig. 1. See list of informatics articles in Appendix B.

After identification, we screened articles based on inclusion and exclusion criteria. We included articles on cognitive decline and impairment and sex-specific studies, healthcare disparities, and informatics literature, published between 2000 and 2025. Only full-length, peer-reviewed articles in English were considered. We excluded articles on genomes and non-human studies, non-peer-reviewed articles, generic surveys and reviews on dementia. During manual screening, we exclude any articles that focus on non-biological disparities, if present. Most of the articles that were manually excluded contained information on sex distribution in the abstract but did not analyze sex-specific disparities. Additionally, some studies were only partially related to cognitive decline, addressing it as a comorbidity within other contexts such as COVID-19.

For study selection, one author (M.G.) extracted key information from all articles, including publication year, geographic location, study characteristics such as clinical conditions, clinical tasks, study size, and role of informatics (as reported in Appendix D), presence of sex-specific findings, relevance to cognitive decline and impairment, and connection to healthcare informatics. The extracted data were reviewed and discussed among three authors (M.G., X.L., J.L.). Two authors (with the third serving as a tiebreaker) reviewed each full-text article for eligibility criteria. A full checklist of the preferred reported items for the PRISMA extension of scoping reviews (PRISMA-ScR) [5] is available in Appendix C, providing detailed guidelines to ensure comprehensive and transparent reporting.

## Results

We present the results in three parts: (i) the metadata analysis to examine the bibliographic landscape of the research field and its development over time, (ii) the attributes of the studies (types, methods, and data) to understand the context in which the studies were conducted, and (iii) thematic analysis in the context of health informatics and science aspects. We provide discussions to reveal gaps in literature and suggest directions for future research.

### Metadata statistics

We identified 17 papers that met our search criteria and analyzed bibliometric information to identify characteristics of articles (Table 2). About 41.17% (*n* = 7) of publications are from North America [10–16], followed by the European Union [17–21]. Majority (*n* = 11) of the publications are single-site studies [11–16, 18, 22–25], while the other multi-site collaborations (*n* = 6) have emerged among hospitals, and academic institutions, research institutions [10, 17, 19–21, 26]. More than half of the eligible studies are published after 2020 (*n* = 10). Most of the studies are published in Springer Nature [16, 18, 19, 26, 27] and JMIR [11, 13, 20]. Three out of 17 studies mentioned about racial demographics out of which two of them mentions the majority as white, non-Hispanic population [11, 12] whereas the third shows majority of the Hispanic population [13]. While other studies do not explicitly mention anything about race, 4 out of 17 other studies considered participants from areas inhabited with West-Eurasian (Caucasoid) [17, 18, 20, 21]. Similarly, a few other studies focussed on Asian population [22, 26]. However, the racial differences of studies on finding sex-specific disparities for cognitive decline and impairment needs further investigation.

### Attributes

We explored three key attributes, such as study types, study methods, and study data, to comprehensively understand the research landscape related to sex-specific disparities. Table 3 contains summaries of all articles we reported.

**Table 2:** Characteristics of included studies in the scoping review

Characteristic	Category	Frequency (%)
Year of Publication	2025	1 (5.9%)
	2024	3 (17.6%)
	2023	4 (23.5%)
	2022	1 (5.9%)
	2021	1 (5.9%)
	2018	2 (11.7%)
	2017	1 (5.9%)
	2016	1 (5.9%)
	2015	1 (5.9%)
	2009	1 (5.9%)
	2000	1 (5.9%)
Geographic region	North America	7 (41.2%)
	South America	1 (5.9%)
	European Union	5 (29.4%)
	Asia	3 (17.6%)
	Australia	1 (5.9%)
Publication house	JMIR Publications	3 (17.6%)
	Springer Nature	5 (29.4%)
	Wiley	2 (11.7%)
	Elsevier	2 (11.7%)
	Others	5 (29.4%)
Care continuum	Identification	7 (41.2%)
	Recovery	5 (23.8%)
	Monitoring	4 (23.5%)
	Prevention	1 (5.9%)
Study method	Statistical Methods	8 (47.1%)
	Machine Learning	4 (23.5%)
	Others	5 (29.4%)

**Table 3:** Summary of attributes of reported studies

Ref	Aim	Study method	Study data (size)	Study types (sex-specific analysis)
***Studies with people having cognitive decline and impairment using machine learning***				
[14]	Identified features associated with conversion from MCI to AD whose temporal evolution significantly differs between men and women	Autoregressive correlation structure	ADNI database. 168 subjects (59 females)	Females may exhibit more pronounced symptoms or a greater degree of cognitive impairment at the onset of the evaluation compared to males. Females experience a faster rate of cognitive impairment per year compared to males
[24]	Investigated health service use for people in the last year of life	Logistic regression modeling	Western Australian data linkage system (27971 persons)	Sex differences were also evident in the finding that males with documented AD were more likely to be hospitalized in their last year of life compared to females with documented AD
[23]	Quantified the severity of AD by exploring complex interactions between features	Multifactor affiliation analysis	Open access series of imaging studies. 354 observations, 142 subjects	Female subjects with less education are more likely to have dementia. Older female subjects with less education and lower socio-economic status are affected by Alzheimer’s disease
[10]	Investigated correlations between brain modalities and AD cohort	Multi-group tensor canonical correlation analysis	ADNI (female: 242 + male: 299 for group 1; female: 162 and male: 200 for group 2)	Identified sex-specific cross-modality imaging correlations, supporting sex differences in brain metabolism in the thalamus and cerebellum regions
[19]	Presented homogeneous clusters of patients	Multi-layer clustering	ADNI (317 females and 342 males)	Two clusters for the female population were compared to four clusters for the male population. Properties of the constructed clusters demonstrate the sex-specific differences for AD patient groups
[17]	Explored the difference in performance of AI compared to traditional statistical techniques for prediction of all-cause dementia	Elastic net Cox regression, support vector machine, random forest	Age, gene/environment susceptibility- Reykjavik study 4893 participants	Removing imaging variables slightly reduced AUC and c-statistics, but values remained high. In elastic net regression, models performed similarly across sexes, whereas in elastic net Cox regression, males showed higher c-statistics than females
[18]	Identified key factors associated with access to assistive technology and telecare technology	Regression models	LIVE@Home.Path trial 276 persons with dementia	Being female, of increasing age, living alone, and with comorbidity and low physical function were key factors for access to assistive technology and telecare
[22]	Constructed a machine learning model predicting the maximum care-needs level for the next 5 years of 75 years old	Multinomial logistic regression	47,862 participants from metropolitan areas of Japan	Females with dementia had a strong influence on diseases related to the musculoskeletal system and connective tissue. Females were reported at increased risk of long-term care needs
***Studies with people who have cognitive decline and impairment using other than machine learning***				
[15]	Addressed the issues for the use of virtual environment (VE) with older adults	VE for 3D space	30 adults	Males demonstrated decreased performance on the mental rotation test with age but still performed at a significantly higher level than older females
[11]	Conducted study to understand the factors related to participation in the health eHeart Study	Remote email recruitment campaign	UCSF patients: registered 7012,consent by 5899	Patients with dementia, however, were less likely to join the study, especially when considering their other health issues (comorbidities). Females were more likely to participate in the study than males
[26]	Explored the effects of 8-week Flexi-bar vibration exercise (FE) on the cognitive function in middle-aged and elderly women	Statistical method	Thirty middle-aged and elderly women	8-week Flexi-bar vibration exercise improve cognitive performance in middle-aged and elderly women
[28]	Compared the increase in AD mortality rate (ADMR) in Brazilian regions over the years 2010 to 2020 with the increase in life expectancy at birth	Statistical method	Brazilian Institute of Geography and Statistics (IBGE), from the Department of Informatics and Technology of the Brazilian Ministry of Health (DATASUS) and from the Brazilian Institute of the Environment and Renewable Natural Resources (IBAMA)	Female population has a higher life expectancy at birth than male
[29]	Data analysis of establishing accurate prevalence rates of dementia and Alzheimer’s disease in Bulgaria	Statistical method	642,013 unique patients (Male 39.88%, Female 60.12%) obtained from National Health Insurance Fund (NHIF)	Compared to male patients, the prevalence rates of female patients begin to increase fast between 65–70, while in the case of Alzheimer’ disease this happens at the age of 55–60
[30]	Characterized the progression of health conditions, such as chronic diseases and neuropsychiatric symptoms, from initial mild cognitive impairment diagnosis to dementia onset	Network Analysis	The participants in Mayo Clinic Study of Aging (MCSA)	Reports increased uncertainty in predicting cognitive status among female participants due to change in chronic conditions
***Studies with caregivers***				
[13]	Developed mHealth interventions for family caregivers of people with dementia	A cross-sectional survey	50 caregivers of people	The study found that there were more female caregivers compared to national data. This could be because Hispanic females in rural areas are more likely than others to care for their families.
[20]	Analyzed interviews from informal caregivers, overburdened or isolated caregivers	Proposed Diapason, automated program	49 caregivers of persons with AD	Female spouses expressed negative (2/3) or neutral opinions (1/3). Daughters expressed more qualified opinions about the program compared to female spouses

#### Study types

Research on sex-specific differences in cognitive decline and impairment has highlighted distinct patterns across multiple dimensions. Interestingly, most studies are experimental studies. Females have been found to exhibit more severe cognitive impairment and experience a faster rate of decline compared to males at the onset of AD [14].

Sociodemographic factors, such as lower education level and socioeconomic status, disproportionately affect older females, increasing their vulnerability to AD and dementia [19, 23]. Furthermore, clustering analyses of AD patient populations demonstrate divergent patterns, with females forming fewer but more defined clusters compared to males, indicating potential variability in disease trajectories [19]. Imaging studies have revealed sex-specific differences in brain metabolism, particularly in the thalamus and cerebellum, supporting biological distinctions in disease progression [10]. A comprehensive controlled study demonstrates that the 8-week Flexi-bar vibration exercise improve cognitive performance in middle-aged and elderly females [30]. Past research suggests potential sex-specific odds that are associated with dementia [12].

Most other studies are observational (n = 8) [11, 13, 15, 17, 18, 20, 22, 24]. The gain in life expectancy at birth observed for females was 2.9 years and for males it was 3.1 years in the period from 2010 to 2020. The female sex presented the biggest Alzheimer’s disease mortality rate in the South and Southeast [28]. Predictive models for cognitive decline and impairment show comparable performance across sexes in some frameworks [15, 17], such as elastic net regression, though males tend to exhibit higher c-statistic values in Cox regression analyses. In terms of healthcare utilization, males with AD are more likely to be hospitalized in their final year of life, while females with cognitive decline and impairment, especially those with comorbidities and lower physical function, are more likely to rely on assistive technology and telecare services [18, 24]. Moreover, females having dementia are less likely to participate in clinical studies, despite being slightly overrepresented in some cohorts, such as the Health eHeart Study [11]. Another study reported increased uncertainty in predicting cognitive status among female participants due to sex-specific differences in (i) chronic conditions, (ii) Cognitive and functional impairment, and (iii) neuropsychiatric characteristics [16]. We find also that, compared to male patients, the dementia prevalence rates of dementia among female patients begin to increase fast between 65–70, while in the case of Alzheimer’ disease this happens at the age of 55–60 [29].

Caregiver studies reflect the sex-specific dynamics in dementia care, with a higher proportion of female caregivers, particularly in Hispanic rural populations [13, 20]. Females having dementia are also at increased risk for long-term care needs. People with dementia and their caregivers—primarily female spouses and daughters—often express varied emotional responses to caregiving programs [22]. Collectively, these findings emphasize the importance of considering sex-specific factors in caregiving factors of cognitive decline and impairment research, which has implications for tailored interventions, healthcare delivery, and support mechanisms across different populations.

Noticeably, 3 out of 17 were pilot studies, carried out in Iceland [17], France [20], and USA [13], respectively. This inclusion of three pilot studies suggests ongoing exploratory efforts to assess feasibility before conducting larger-scale research. Among 17, five are cross-sectional studies [10, 13, 15, 20, 24], seven of them are retrospective longitudinal studies [11, 12, 14, 17, 19, 22, 23], and only one is a prospective longitudinal study [18]. This trend suggests a focus on capturing data at a single point in time or analyzing the past records rather than tracking participants over time. The presence of only one prospective longitudinal study highlights a potential gap in long-term observational research.

The care continuum in the studies predominantly focus on identification [10, 12, 14, 19, 23] and monitoring [17, 22, 24], indicating a strong emphasis on recognizing biomarkers, associations, and patterns related to dementia and cognitive decline. Several studies address recovery [13, 15, 18, 20], particularly by sex-specific performance differences, suggesting an interest in post-diagnosis management and rehabilitation. However, prevention is minimally represented [11], highlighting a potential gap in proactive, risk-reduction research. The focus on monitoring and identification reflects the current research trend towards early detection and progression tracking rather than intervention or prevention strategies. Interestingly, we found no informatics-based studies addressing sex-specific disparities in treatments or procedures for cognitive decline and impairment.

#### Study methods

This section summarizes the approaches in reviewed articles to improve the understanding, diagnosis, and management of AD, dementia, and other related conditions, with particular emphasis on addressing sex-specific differences. From encompassing the need of care for individuals with dementia [13, 22, 24] to behavioral analysis with the decision-making [11, 20, 30], the informatics literature has limited contribution toward sex-specific treatment plans for people with dementia. In their efforts, they draw attention toward the equitable care-need levels by caregivers, nursing, medical, and allied health services.

We also identified studies using descriptive analysis [19, 23, 29, 31] and predictive analytics [14, 17, 30] for individuals with mild cognitive impairment (MCI) or dementia. Following their examination of cohort characterization and dementia prediction, the studies underscore the need for evidence-based quantification of sex-specific disparities in healthcare.

While identifying risk factors associated with cognitive decline and impairment has gained significant attention in recent years, only four studies have specifically focused on informatics-based analyses of risk factors for Alzheimer’s disease and related dementia among males and females [10, 12, 31, 32]. We also observed the technological advancements supporting neuropsychological tests [15, 16] and telecare for home-dwelling individuals with dementia [18]. These findings highlight the unmet potential for reliable communication and sensing technologies for this population.

The analysis reveals that a mix of statistical methods and machine learning techniques were used across studies (see Table 2). Statistical methods, including tensor canonical correlation analysis, regression models, autoregressive correlation structures, and affiliation analysis, were frequently employed for pattern identification and sex-specific characterization [10, 11, 14, 18, 23]. Machine learning techniques such as elastic net regression, random forest, support vector machines, multinomial logistic regression, clustering, and logistic regression modeling were employed for predicting cognitive decline and analyzing sex-specific differences [17, 19, 22, 24]. One out of 17 studies used network analysis to gauge the changes in progression of cognitive impairment [16]. Notably, some other studies used assessments, questionnaires, and mobile devices for data collection mechanisms to perform observational studies [13, 15, 20].

#### Study data

The reviewed studies used various types of data including multisite study data (Alzheimer’s Disease Neuroimaging Initiative [ADNI]) which is a series of imaging and clinical data designed to enhance research focused on AD diagnosis and progression [10, 14, 19]. The dataset includes various imaging modalities, such as structural MRI, functional MRI, and PET scans, alongside cerebrospinal fluid biomarkers and comprehensive neuropsychological test scores [33, 34].

The size of study data used in the reviewed articles varies ranging from < 30 [13, 20, 24, 35] to >10,000 subjects [11, 29]. Analytical techniques used moderate sample sizes to enable subgroup analysis based on sex and race [10, 16, 32]. Risk factor analysis, descriptive and predictive modeling studies utilize larger datasets, divided into training and test sets, to enhance the robustness of the models and their applicability to diverse populations [12, 14, 15, 17, 19, 22]. Recruitment studies often involve extensive data collection efforts, exemplified by large-scale email campaigns that engage thousands of participants [11, 31]. Observational analysis with large datasets enhances the reliability of evidence-based findings in informatics literature. Nevertheless, establishing frameworks for sex-specific analyses has been achieved with smaller datasets, contingent upon the study design [13, 35].

### Thematic analysis

To further examine the intersection of health informatics and science aspects on sex differences, we classified the articles thematically in Table 4.

**Table 4:** Thematic classification of articles.

	[10]	[23]	[20]	[24]	[15]	[17]	[11]	[22]	[12]	[13]	[18]	[14]	[19]	[31]	[28]	[29]	[30]
Healthcare informatics																	
CDSS		X					X	X	X		X	X	X		X	X	
AI	X			X	X	X		X					X	X			X
TTM			X							X	X						
Science aspect																	
Clinical					X	X		X	X	X		X			X	X	X
Biomed	X	X										X	X				
Psych & Behav			X	X			X				X			X			

CDSS: Clinical Decision Support Systems; AI: Artificial Intelligence, health analytics, big data; TTM: Telemedicine, Telehealth, and Mobile health (mHealth) apps; Clinical: Sex differences in cognitive decline and impairment patterns and assessment; Biomedical: Sex differences in pharmacological and neuroimaging studies; Psychosocial & Behavioral: Sex differences in digital health, behavioral, and psychosocial, demographic factors

In health informatics, Clinical Decision Support Systems (CDSS) is being actively explored for its integration with other advanced technologies like AI and health analytics [19, 22, 36]. The interest in CDSS suggests a growing recognition of the need for systems that can assist clinicians in making better-informed decisions, in patients with cognitive decline and impairment, considering sex-specific differences. AI is a major focus area, particularly in its potential to revolutionize healthcare through predictive analytics and big data analysis. The intersection with CDSS suggests that AI is integrated into decision support frameworks. The focus on Telemedicine, Telehealth, and Mobile Health (mHealth) Apps (TTM) indicates a shift towards digital health solutions, especially important in a post-pandemic world and when considering mobility impairment of elderly persons where remote healthcare has become essential [16]. The integration of TTM with sex-specific differences research suggests that digital health platforms are being studied for their effectiveness across different sex demographics.

Sex differences play a crucial role across various scientific aspects such as clinical, biomedical, and psychosocial & behavioral analysis for developing sex-specific interventions and treatment protocols. Clinically, they influence patterns of cognitive decline and impairment, as well as assessment strategies. In biomedical studies, these differences are evident in pharmacological responses and neuroimaging findings. Additionally, psychosocial and behavioral factors, including digital health engagement, behavioral patterns, and demographic influences, also exhibit notable sex-based variations. Studies on how pharmacological treatments and neuroimaging results differ between sexes could lead to more effective and personalized drug therapies and a better understanding of neurodegenerative diseases. Research in this area highlights the importance of considering sex differences in digital health adoption, user behavior, and psychosocial factors.

## Discussion

Studies in sex differences in cognitive decline and impairment remain limited in the informatics literature, despite the vast increase in digitization of EHRs since 1992. Most studies are patient-centric whereas only a few of them are associated with caregivers’ experience. We did not observe any direct similarities or differences among studies conducted in North America (including US) and the European Union.

We identified three aspects of sex-specific differences in reported studies, namely, (i) Clinical: cognitive decline and impairment patterns and assessment, (ii) Biomed: pharmacological and neuroimaging studies, and (iii) Psycho & Behav: behavioral, psychosocial, and demographic factors. The key observations are summarized as follows:

1. Clinical: Evidence indicates clear sex-specific disparities in dementia diagnosis and progression. Studies report that the rate of cognitive impairment and mortality associated with Alzheimer’s disease significantly differ between males and females. Additionally, predictive models for dementia also exhibit variation across sexes, underscoring the need for sex-stratified analyses in clinical assessments.
2. Biomedical: Sex differences are evident in neurobiological and cognitive domains, including performance on mental rotation tests and variations in brain metabolism within the thalamus and cerebellum. Females demonstrate a faster annual rate of cognitive decline than males, though it remains uncertain whether this difference is rooted in biological factors. Cluster analyses based on biomedical features further reveal distinct sex-specific patterns among Alzheimer’s disease cohorts.
3. Psychological and Behavioral: Research highlights sex-based disparities in both the care requirements of individuals with cognitive impairment and the demographic composition of their caregivers, emphasizing the psychosocial dimensions of sex differences in dementia care.

While dementia remains untreatable, differences in treatment approaches and procedures may exist between males and females, underscoring the need for personalized medicine for delayed dementia progression and better quality of life. Therefore, it is essential to explore treatment accessibility, therapeutic efficacy, and care outcomes to identify sex-specific differ in treatment-based informatics for dementia. A future study could explore modifiable sex-specific risk factors and comorbidities that may contribute to delaying cognitive decline and impairment, building upon previous research [36, 37]. Although clinical, biomedical, psychological and behavioral sex-specific differences exist at various care continuum, gaps remain in informatics literature. Addressing these gaps by incorporating sex-specific considerations in dementia diagnosis and tracking disease progression from normal cognition to dementia holds great potential for improving patient outcomes, as evidenced by recent studies.

Studies employ statistical methods, machine learning techniques, and descriptive analyses to examine sex differences, using public data, imaging data, and recruitment data with sample sizes ranging from small (*n* < 30 subjects) to large (*n* >10,000 subjects). CDSS increasingly requires AI-driven solutions to aid clinicians in making informed decisions for personalized treatments that consider sex differences [38, 39]. Additionally, intrinsic and extrinsic explainable models within CDSS may contribute towards human-understandable decision-making. Telemedicine and mobile health technologies have been investigated for identifying sex differences; however, existing evidence remains insufficient for their widespread integration into real-time clinical practice.

**Fig. 2:**
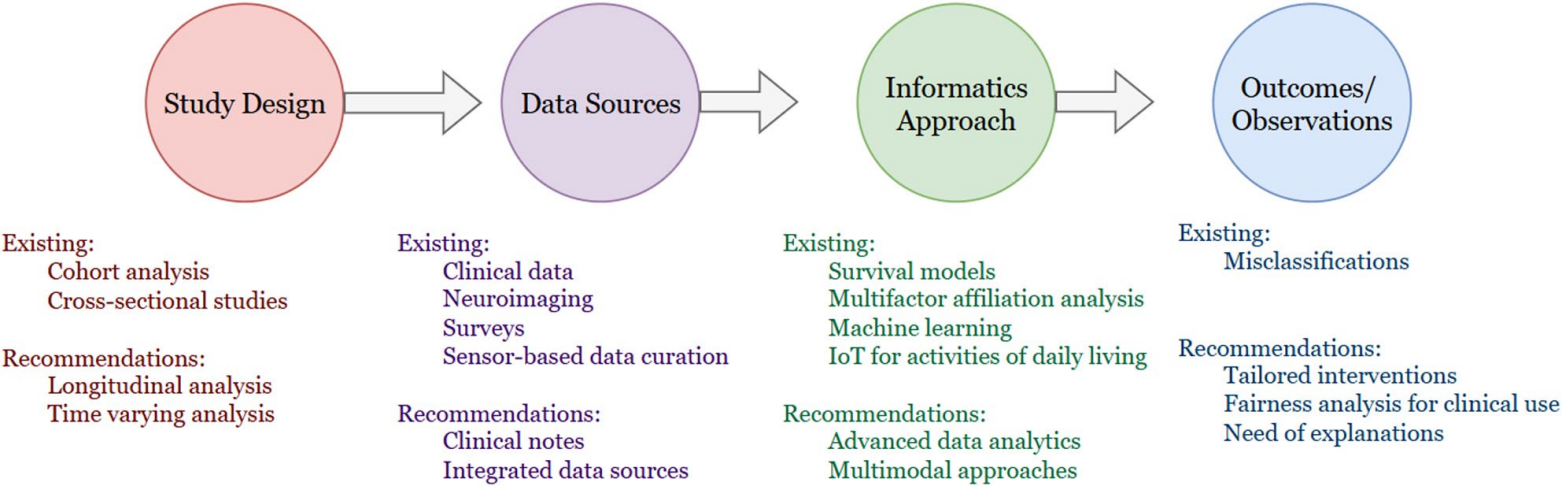
Existing studies and recommendations for healthcare informatics research on sex-specific differences in cognitive decline and impairment. Here, IoT is Internet of Things

The current scope of sex difference research lacks informatics studies examining the influence of lifestyle factors and environmental exposures on the onset and progression of cognitive decline (i.e., cognitive concerns) and impairment (i.e., MCI, dementia) that could provide a deeper understanding of sex differences. Furthermore, considering how sex-specific differences intersect with other demographic factors—such as race, ethnicity, and socioeconomic status—could help elucidate their compounded effects on cognitive impairment risk and progression [40]. Advancing and evaluating technologies such as wearable devices and mobile health applications could enhance the monitoring and management of AD and dementia in a manner that accounts for sex-specific differences. We summarized the study design, data sources, informatics approach and outcomes in the reported studies and made recommendations to facilitate future research (see Fig. 2).

First, *longitudinal studies*: conducting large-scale, long-term studies will be invaluable in tracking sex-specific disease trajectories from early cognitive decline to advanced dementia. These studies can help identify critical windows for intervention and prevention. Second, *integrated data source*s *(multimodal data)*: combining multiple data sources, including EHRs, wearable devices, and patient-reported outcomes, will provide a more comprehensive assessment of patients’ health and cognitive status. Third, *advanced data analytics*: utilizing advanced machine learning techniques using multimodal data can help uncover patterns and predictors that traditional approaches may overlook. The insights gained through these analytics may lead to new advancements in dementia research. Finally, *tailored intervention*: designing and testing sex-specific interventions is crucial for optimizing treatment outcomes and enhancing patient care. By addressing these areas, informatics can play a pivotal role in bridging gaps in sex disparity research, ultimately leading to more personalized and effective strategies for managing cognitive impairment and dementia.

Our scoping review has several limitations. The number of studies included is relatively small. Our search strategy was limited to informatics literature on sex-specific differences and disparities in cognitive decline and impairment, which do not cover the full breath of sex-specific studies in the boarder clinical literature. The discipline of informatics primary focused on informatics journals and conferences alone may not fully represent its scope. However, this scope enables a more focused assessment of data-driven methodologies highlighting opportunities for advancing sex-aware models and algorithms in informatics cognitive decline and impairment research. Although the manuscripts included in our scoping review do not cover exhaustive sex-specific studies in the boarder clinical literature, our review offers a valuable synthesis of how informatics and clinical researchers have begun to engage with this topic and where opportunities exist for future work.

## Conclusion

This scoping review summarizes the use of health informatics, particularly in neuroimaging, machine learning applications, and cognitive assessment tools to understand sex differences in cognitive decline and impairment. Findings from these studies may contribute to an early diagnosis and management of neurodegenerative diseases such as AD and related dementias by identifying key differences between males and females. The review reveals potential research directions in sex-specific differences in healthcare, emphasizing the need for tailored interventions and equitable access to resources. Despite the increasing use of informatics in the field, research gaps persist, underscoring the necessity for further research, validation, and comprehensive approaches to enhance healthcare delivery and outcomes for both males and females.

## Supplementary Information

Supplementary Material 1

Supplementary Material 2

Supplementary Material 3

Supplementary Material 4

## Data Availability

No datasets were generated or analysed during the current study.

## Footnotes

1
https://www.alzheimersresearchuk.org/.
