# Supplementary material for: Sex differences in cognitive decline and impairment: a scoping review in informatics literature
Source: Biol Sex Differ. 2025 Dec 16;17:11. doi: 10.1186/s13293-025-00804-6 (PMC12822129; doi:10.1186/s13293-025-00804-6)
Supplement: Supplementary file 1 — Supplementary Material 1 [file 13293_2025_804_MOESM1_ESM.docx]

Appendix A

Table A1: Concepts for search strategy

|  | **Concept** | **Search Strings** |
| --- | --- | --- |
| A | Cognitive decline and impairment | *(((cognitive or cognition) or (disorder* or defect* or disabilit* or dysfunction* or impairment* or declin* or MCI or CI)) or Alzheimer* or dementia* or "executive dysfunction*" or "Frontotemporal Lobar Degeneration" or "Lewy Body Disease" or "mental deterioration" or "mental deteriorations" or "Primary Progressive aphasia*")* |
| B | Sex-specific terms | *(female or females or "gender-specific" or "sex-specific" or woman or women)* |
| C | Healthcare disparities terms | *(difference* or disparit* or equalit* or equit* or factor or factors or inequalit* or inequit*)* |
| D | Informatics literature terms | *(bioinformatics or "computer science*" or informatics or "information science*" or "information technologies" or "information technology")* |
| E | Genetics and proteomics terms | (gene or genes or genetic* or genomic* or metabolomic* or molecular or RNA).ti,ab. |
| F | Animal related studies | ((alpaca or alpacas or amphibian or amphibians or animal or animals or antelope or armadillo or armadillos or avian or baboon or baboons or beagle or beagles or bee or bees or bird or birds or bison or bovine or buffalo or buffaloes or buffalos or "c elegans" or "Caenorhabditis elegans" or camel or camels or canine or canines or carp or cats or cattle or chick or chicken or chickens or chicks or chimp 11516646  or chimpanze or chimpanzees or chimps or cow or cows or "D melanogaster" or "dairy calf" or "dairy calves" or deer or dog or dogs or donkey or donkeys or drosophila or "Drosophila melanogaster" or duck or duckling or ducklings or ducks or equid or equids or equine or equines or feline or felines or ferret or ferrets or finch or finches or fish or flatworm or flatworms or fox or foxes or frog or frogs or "fruit flies" or "fruit fly" or "G mellonella" or "Galleria mellonella" or geese or gerbil or gerbils or goat or goats or goose or gorilla or gorillas or hamster or hamsters or hare or hares or heifer or heifers or horse or horses or insect or insects or jellyfish or kangaroo or kangaroos or kitten or kittens or lagomorph or lagomorphs or lamb or lambs or lemur or lemurs or llama or llamas or macaque or macaques or macaw or macaws or marmoset or marmosets or mice or minipig or minipigs or mink or minks or monkey or monkeys or mouse or mule or mules or nematode or nematodes or octopus or octopuses or orangutan or "orang-utan" or orangutans or "orang-utans" or ostrich or ostriches or oxen or parrot or parrots or pig or pigeon or pigeons or piglet or piglets or pigs or porcine or primate or primates or quail or rabbit or rabbits or rat or rats or reptile or reptiles or rodent or rodents or ruminant or ruminants or salmon or sheep or shrimp or slug or slugs or swine or tamarin or tamarins or toad or toads or trout or urchin or urchins or vole or voles or waxworm or waxworms or wildlife or worm or worms or xenopus or "zebra fish" or zebrafish) not (human or humans or patient or patients)).ti,ab,hw,kf. |
| G | Non-peer-reviewed studies | limit 22 to (dissertation abstract or conference abstract or book or chapter or editorial or erratum or note or addresses or autobiography or bibliography or biography or blogs or comment or dictionary or directory or interactive tutorial or interview or lectures or legal cases or legislation or news or newspaper article or overall or patient education handout or periodical index or portraits or published erratum or video-audio media or webcasts or clinical trial protocol) [Limit not valid in APA PsycInfo,CCTR,CDSR,Embase,Ovid MEDLINE(R); records were retained] |

* means “any possible ending after this root word.”
