# Supplementary material for: Sex differences in cognitive decline and impairment: a scoping review in informatics literature
Source: Biol Sex Differ. 2025 Dec 16;17:11. doi: 10.1186/s13293-025-00804-6 (PMC12822129; doi:10.1186/s13293-025-00804-6)
Supplement: Supplementary file 2 — Supplementary Material 2 [file 13293_2025_804_MOESM2_ESM.docx]

**Appendix A: Titles of all journals and conference proceedings covered by the search.**

1. AMIA Annual Symposium Proceedings

2. AMIA Summits on Translational Science Proceedings

3. Applied Clinical Informatics

4. Applied Informatics

5. Applied Medical Informatics

6. Artificial intelligence in Medicine

7. Biomedical Informatics Insights

8. BMC Medical Informatics & Decision Making

9. BMJ Health & Care Informatics

10. Brain Informatics

11. Brain Informatics and Health: International Conference Proceedings

12. Cancer informatics

13. CIN: Computers, Informatics, Nursing

14. Computing and Informatics

15. Critical Reviews in Medical Informatics

16. Earth Science Informatics

17. Ecological Informatics

18. European Journal for Biomedical Informatics

19. Genome Informatics

20. Genomics & Informatics

21. Health Communications and Informatics

22. Health Informatics Journal

23. Healthcare Informatics

24. Healthcare Informatics Research

25. Proceedings of the International Conference on Health Informatics and Medical Systems

26. The IEEE Intelligent Informatics Bulletin

27. IEEE International Conference on Healthcare Informatics

28. IEEE Journal of Biomedical and Health Informatics

29. IEEE Transactions on Industrial Informatics

30. Indian Journal of Medical Informatics

31. Informatics

32. Informatics for Health & Social care

33. Informatics in Medicine Unlocked

34. Informatics in Pathology

35. Informatics in Primary Care

36. International Journal of Functional Informatics & Personalised Medicine

37. International Journal of Healthcare Information Systems & Informatics

38. International Journal of Medical Engineering and Informatics

39. International Journal of Medical Informatics

40. Japan Journal of Medical Informatics

41. JCO Clinical Cancer Informatics

42. JMIR Medical Informatics

43. Journal of Biomedical Engineering & Informatics

44. Journal of Biomedical Informatics

45. Journal of Health & Medical Informatics

46. Journal of Health Informatics

47. Journal of Health Informatics in Developing Countries

48. Journal of Healthcare Informatics Research

49. Journal of Informatics Nursing

50. Journal of Innovation in Health Informatics

51. Journal of Medical Imaging & Health Informatics

52. Journal of Medical Internet Research

53. Journal of Medical Statistics & Informatics

54. Journal of Ocular Biology, Diseases, and Informatics

55. Journal of Pathology Informatics

56. Journal of the American Medical Informatics Association

57. Journal on Systemics Cybernetics & Informatics

58. LIPICS: Leibniz International Proceedings in Informatics

59. Medical Informatics

60. Medical Informatics & the Internet in Medicine

61. Medical Informatics and the Internet in Medicine

62. Methods of Information in Medicine

63. Microbial Informatics and Experimentation

64. Molecular Informatics

65. Network Modeling Analysis in Health Informatics and Bioinformatics

66. Proceedings of the International Congress on Nursing Informatics

67. Online Journal of Nursing Informatics

68. Online Journal of Public Health Informatics

69. The Open Medical Informatics Journal

70. Proceedings of the International Workshop on Social Informatics

71. Proceedings of the ACM International Workshop on Data and Text Mining in Biomedical Informatics

72. Proceedings of the ACM Conference on Bioinformatics, Computational Biology and Biomedical Informatics

73. Proceedings of the IEEE International Conference on Progress in Informatics & Computing

74. Proceedings of the International Conference on Biomedical Engineering & Informatics

75. Security Informatics

76. Social informatics: the International Conference Proceedings

77. Studies in Health Technology & Informatics

78. Yearbook of Medical Informatics
