# Supplementary material for: Sex differences in cognitive decline and impairment: a scoping review in informatics literature
Source: Biol Sex Differ. 2025 Dec 16;17:11. doi: 10.1186/s13293-025-00804-6 (PMC12822129; doi:10.1186/s13293-025-00804-6)
Supplement: Supplementary file 3 — Supplementary Material 3 [file 13293_2025_804_MOESM3_ESM.docx]

| SN | Title | Link | DOI | Year | Journal/ Conference | Country | Sex-specific analysis | Cognitive Impairment | healthcare informatics | Publisher | Citation count | Journal h5-index |
| --- | --- | --- | --- | --- | --- | --- | --- | --- | --- | --- | --- | --- |
| 1 | Multi-Group Tensor Canonical Correlation Analysis | <https://dl.acm.org/doi/abs/10.1145/3584371.3612962> | 10.1145/3584371.3612962 | 2023 | Proceedings of the 14th ACM International Conference on Bioinformatics, Computational Biology, and Health Informatics | USA | Yes | Yes | Yes | Association for Computing Machinery | 0 | 27 |
| 2 | Dementia prediction in the general population using clinically accessible variables: a proof-of-concept study using machine learning. The AGES-Reykjavik study. | <https://link.springer.com/content/pdf/10.1186/s12911-023-02244-x.pdf> | <https://dx.doi.org/10.1186/s12911-023-02244-x> | 2023 | BMC medical informatics and decision making | European Union | Yes | Yes | Yes | Springer | 2 | 59 |
| 3 | Email-Based Recruitment Into the Health eHeart Study: Cohort Analysis of Invited Eligible Patients | <https://www.jmir.org/2023/1/e51238/> | [doi: 10.2196/51238](https://doi.org/10.2196/51238) | 2023 | Journal of Medical Internet Research | USA | Yes | Yes | Yes | JMIR Publications | 0 | 146 |
| 4 | Care-needs level prediction for elderly long-term care using insurance claims data. | <https://www.sciencedirect.com/science/article/pii/S2352914823001673> | <https://www.sciencedirect.com/science/article/pii/S2352914823001673> | 2023 | Informatics in Medicine Unlocked | Japan | Yes | Yes | Yes | Elsevier | 1 | 58 |
| 5 | Risk factors for the development of acute pyelonephritis in women with a positive urine culture. | <https://onlinelibrary.wiley.com/doi/full/10.1002/nau.25005?casa_token=yXc2voDgM-sAAAAA%3AnmeXLd02Gge-TNm1nFA8LrNVJM3jN7dEApqX8PuiBvpaPRMlH5CiD6jTVhpnGxn1gyy9YqTaCFZSD6A> | <https://doi.org/10.1002/nau.25005> | 2022 | Neurourology and Urodynamics | USA | Yes | Yes | Yes | Wiley | 7 | 44 |
| 6 | Factors associated with access to assistive technology and telecare in home-dwelling people with dementia: baseline data from the LIVE@Home.Path trial. | <https://bmcmedinformdecismak.biomedcentral.com/articles/10.1186/s12911-021-01627-2> | <https://doi.org/10.1186/s12911-021-01627-2> | 2021 | BMC Medical Informatics and Decision Making | European Union | Yes | Yes | Yes | Springer | 8 | 59 |
| 7 | Mobile Health, Information Preferences, and Surrogate Decision-Making Preferences of Family Caregivers of People With Dementia in Rural Hispanic Communities: Cross-Sectional Questionnaire Study | <https://www.jmir.org/2018/12/e11682/> | [doi: 10.2196/11682](https://doi.org/10.2196/11682) | 2018 | Journal of medical Internet research | USA | Yes | Yes | Yes | JMIR Publications | 19 | 146 |
| 8 | Longitudinal gender-specific differences in the conversion from mild cognitive impairment to Alzheimer’s disease | <https://ieeexplore.ieee.org/abstract/document/8333404> | DOI: 10.1109/BHI.2018.8333404 | 2018 | In 2018 IEEE EMBS International Conference on Biomedical & Health Informatics (BHI) | Mexico | Yes | Yes | Yes | IEEE Xplore | 4 | 27 |
| 9 | Devising an interpretable calibrated scale to quantitatively assess the dementia stage of subjects with alzheimer’s disease: A machine learning approach. | <https://www.sciencedirect.com/science/article/pii/S2352914816300491> | <https://doi.org/10.1016/j.imu.2016.12.004> | 2017 | Informatics in Medicine Unlocked | India | Yes | Yes | Yes | Elsevier | 13 | 58 |
| 10 | Clusters of male and female Alzheimer’s disease patients in the Alzheimer’s Disease Neuroimaging Initiative (ADNI) database. | <https://link.springer.com/article/10.1007/s40708-016-0035-5> | <https://doi.org/10.1007/s40708-016-0035-5> | 2016 | Brain informatics | European Union | Yes | Yes | Yes | Springer | 32 | 23 |
| 11 | A web-based psychoeducational program for informal caregivers of patients with Alzheimer’s disease: a pilot randomized controlled trial. | <https://www.jmir.org/2015/5/e117/> | doi: 10.2196/jmir.3717 | 2015 | Journal of medical Internet research | European Union | Yes | Yes | Yes | JMIR Publications | 204 | 146 |
| 12 | A population-based retrospective cohort study comparing care for Western Australians with and without Alzheimer’s disease in the last year of life. | <https://onlinelibrary.wiley.com/doi/pdf/10.1111/j.1365-2524.2008.00795.x> | <https://doi.org/10.1111/j.1365-2524.2008.00795.x> | 2009 | Health & social care in the community | Australia | Yes | Yes | Yes | Wiley | 21 | 51 |
| 13 | Issues for the assessment of visuospatial skills in older adults using virtual environment technology. | <https://www.liebertpub.com/doi/abs/10.1089/10949310050078931> | <https://dx.doi.org/10.1089/10949310050078931> | 2000 | CyberPsychology & Behavior | USA | Yes | Yes | Yes | Independent Publisher | 56 | - |
| 14 | Eight-week Flexi-bar vibration exercise enhances cognitive function via altering the circulating levels of BDNF, TNF-alpha, and microRNAs in middle-aged and elderly women. | <https://pubmed.ncbi.nlm.nih.gov/40965604/> | [10.1007/s00421-025-05992-y](https://doi.org/10.1007/s00421-025-05992-y) | 2025 | European Journal of Applied Physiology | China | Yes | Yes | Yes | Springer Nature | 0 | 72 |
| 15 | Alzheimer’s Disease Mortality Rate: Correlation with Socio-Economic and Environmental Factors | <https://pubmed.ncbi.nlm.nih.gov/39195688/> | 10.3390/toxics12080586 | 2024 | Toxics | Brazil | Yes | Yes | Yes | MDPI | 0 | - |
| 16 | Dementia and Alzheimer’s Disease Prevalence in Bulgaria During 2018: Nationally Representative Study | <https://ebooks.iospress.nl/doi/10.3233/SHTI240394> | doi/10.3233/SHTI240394 | 2024 | IOS Press | Bulgaria | Yes | Yes | Yes | IOS Press | 0 | - |
| 17 | Characterizing the progression from mild cognitive impairment to dementia: a network analysis of longitudinal clinical visits | <https://bmcmedinformdecismak.biomedcentral.com/articles/10.1186/s12911-024-02711-z> | 10.1186/s12911-024-02711-z | 2024 | BMC Medical Informatics and Decision Making | USA | Yes | Yes | Yes | Springer Nature | 0 | 116 |
